# A Case of Behçet’s Disease Initially Presenting With Acute Dyspnea Due to Subglottitis

**DOI:** 10.7759/cureus.87684

**Published:** 2025-07-10

**Authors:** Kanako Saigo, Atsunobu Tsunoda, Yuri Ikeda, Masaki Nojima, Toshiharu Matsumoto

**Affiliations:** 1 Otolaryngology - Head and Neck Surgery, Juntendo University, Nerima Hospital, Tokyo, JPN; 2 Dermatology, Juntendo University, Nerima Hospital, Tokyo, JPN; 3 Rheumatology, Juntendo University, Nerima Hospital, Tokyo, JPN; 4 Pathology, Juntendo University, Nerima Hospital, Tokyo, JPN

**Keywords:** apremilast, behçet’s disease, dyspnea, endoscopy, larynx, pseudocroup, subglottic stenosis

## Abstract

We report the case of a 17-year-old female patient who was referred to our department for acute dyspnea. She had noticed erythema all over her body and suddenly noticed severe hoarseness, cough, and wheezing three days before her presentation. Fiberscopy revealed subglottic swelling and aphthae. Subglottitis was diagnosed and intravenous corticosteroids and antibiotics were administered. Subsequently, oral ulcers, genital ulcers, and systemic papules were noted. Histologic examination of the skin suggested Behçet’s disease. Behçet’s disease is an inflammatory disorder that can affect multiple areas of the body, including the mucous membranes of the mouth and genital region, eyes, skin, and joints. To our knowledge, acute dyspnea due to subglottitis has never been reported in patients with Behçet’s disease.

## Introduction

Subglottitis is an inflammation of the infraglottic space [1]. Infection-induced subglottitis is commonly referred to as croup. Croup is classified into true croup, typically caused by diphtheria, and pseudocroup, which is usually caused by viral infections. The most frequent form of subglottitis is pseudocroup, commonly seen in children and caused by viruses. Its main symptoms include a barking cough, hoarseness, and inspiratory stridor. Non-infectious subglottitis is less common, with causes such as trauma, allergic reactions, and burns [2,3]. This report presents a rare case of Behçet’s disease (BD) associated with subglottitis, resulting in acute dyspnea.

## Case presentation

A previously healthy 17-year-old female was admitted to our hospital because of dyspnea. One week before her admission, she noticed erythema on her whole body, measuring approximately 1 cm in diameter. Then, three days before her visit, she developed cough, hoarseness, and sore throat. She complained of dyspnea in the early morning and was taken to the emergency department.

**Figure 1:**
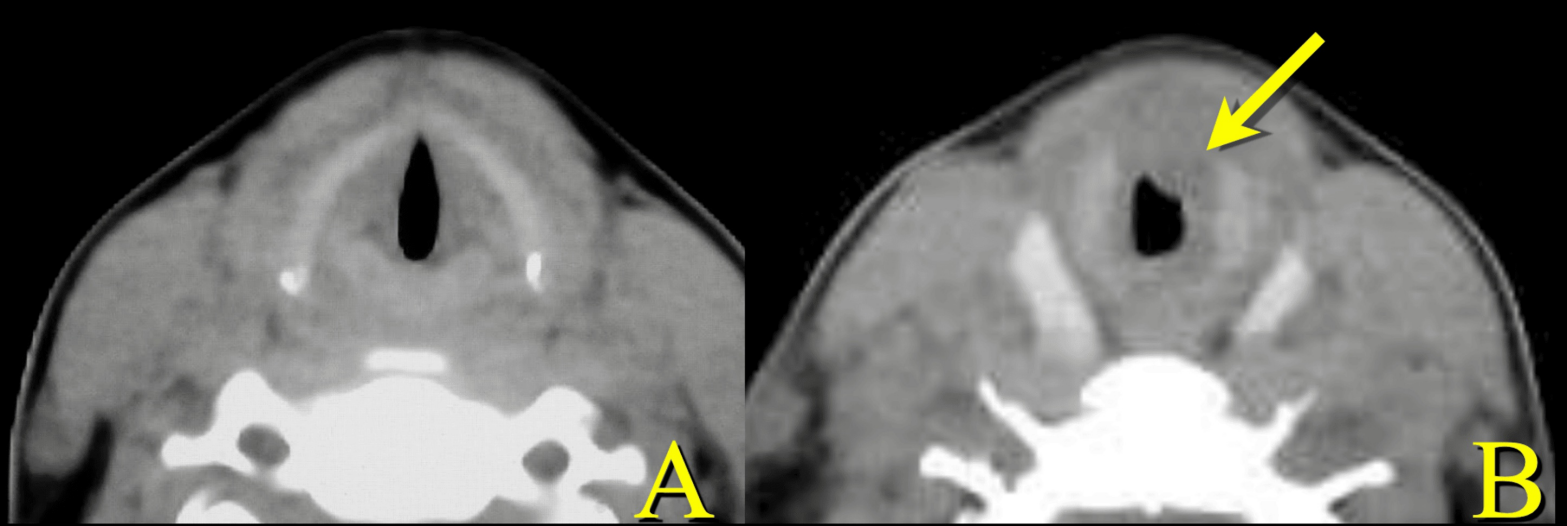
Axial CT at the initial presentation. The glottis appears intact (A). A slightly caudal section (B). Thickening of the anterior tracheal wall (arrow) suggests subglottic stenosis (B).

On presentation, she had a fever of 39.6°C and severe cough and wheezing. Her respiratory rate was 36 breaths/minute and oxygen saturation was 93% on room air. Blood work revealed a white blood cell elevation of 14,900/µL and a C-reactive protein elevation of 6.17 mg/dL, but no other significant findings. CT showed thickening of the upper tracheal wall and subglottic stenosis (Figure 1). She was referred to the otolaryngology department.

**Figure 2:**
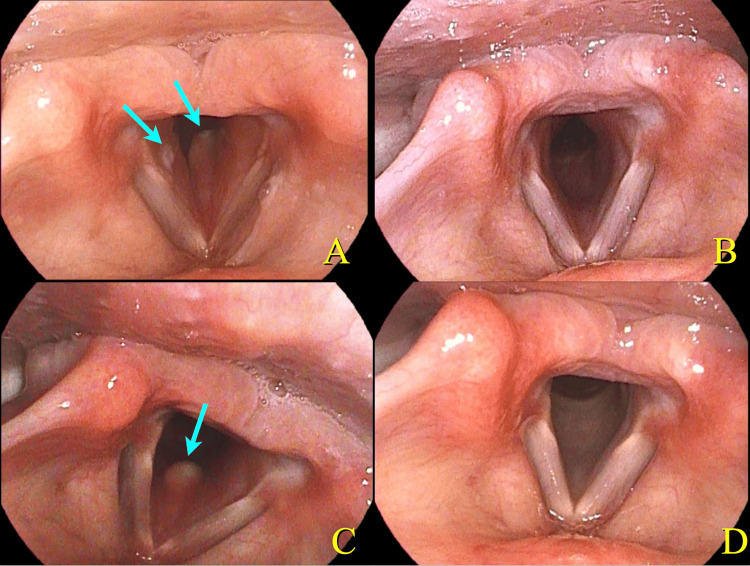
Laryngeal endoscopic findings. A laryngeal image (A) shows bilateral subglottic swelling and aphthae (arrow). Subglottic swelling relieved after the administration of prednisolone (B). Eight months after the onset of symptoms, the patient developed mild dyspnea and showed recurrence of subglottic swelling and aphthae (arrow) (C). She was subsequently treated with apremilast 60 mg daily. A recent laryngeal image shows normal findings (D).

Laryngeal fiberscopy revealed bilateral subglottic swelling and aphthae (Figure 2, Panel A) representing subglottitis.

**Figure 3:**
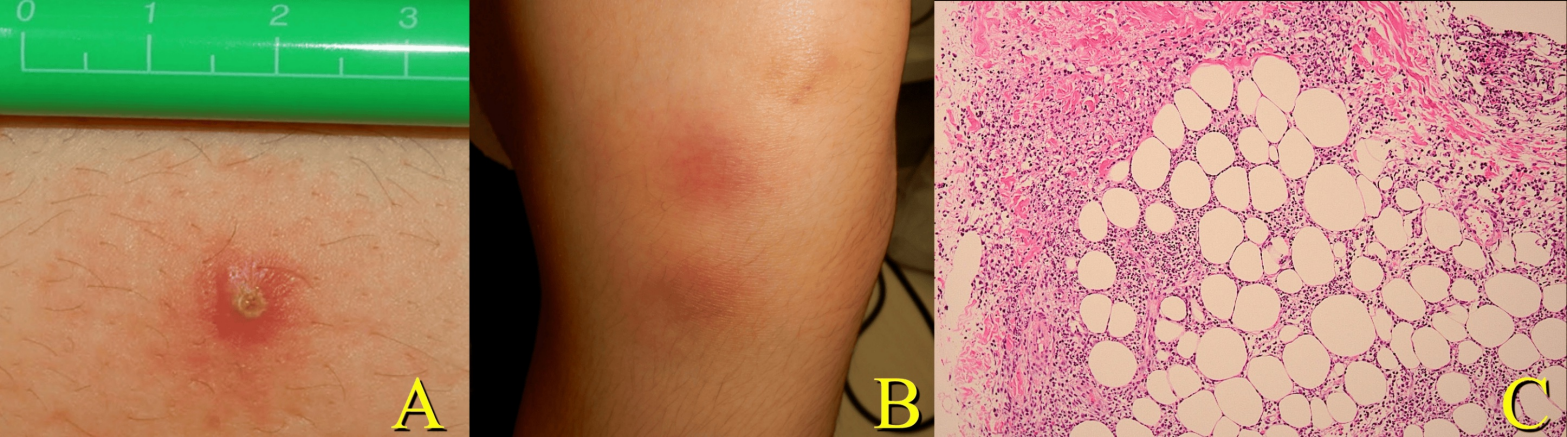
Skin lesions. A red papule with a central pustule was observed on the left thigh (A), and erythematous nodules and plaques were present on the extensor aspect of the left lower leg (B). Histology of the erythema (hematoxylin and eosin, ×100) showed septal panniculitis with neutrophilic infiltration (C). The histopathological findings supported the diagnosis.

A small papule with surrounding erythema was observed on the left thigh (Figure 3A). A systemic disease was suspected, and a dermatologist was consulted. Dermatologic evaluation revealed painless latent oral aphthae, with similar papules on the extremities. Erythema was observed on the extensor aspect of the left lower leg and an ulcer on the left vulva (Figure 3B). Based on the similarity of the other skin and mucosal lesions, a specific systemic disease could cause this subglottic lesion. Skin biopsy of the erythema on the left lower leg showed septal panniculitis with neutrophilic infiltration. Leukocytoclastic and lymphocytic vasculitis were not present in the small vessels (Figure 3C).

To relieve the acute dyspnea, prednisolone 60 mg was administered on the first day, titrated down by 10 mg on the following day for a total of 210 mg. The next day, the fever and dyspnea ceased, and the inflammatory response decreased. Two days after steroid administration, some of the aphthae had disappeared and the subglottic swelling had improved. The patient was discharged from the hospital on day 12 without recurrence of dyspnea, fever, or rash. As the symptoms subsided after treatment, no additional therapy was administered, and the patient was followed up without intervention (Figure 2, Panel B).Two months after discharge from the hospital, she had a recurrence of fever with papules on the lower extremities. According to Japanese diagnostic criteria, this led to the diagnosis of incomplete BD. However, based on International Team for the International Criteria for Behçet’s disease, this case would be diagnosed as BD. Because the recurrence and skin pathology results were consistent with BD, treatment was initiated. Colchicine 1 mg/day was started; however, eight months after the onset of symptoms, the patient developed mild dyspnea and showed recurrence of subglottic swelling (Figure 2, Panel C). Prednisolone 40 mg was administered for five days, followed by 20 mg for five days. Her acute symptoms relieved. She was subsequently treated with apremilast 60 mg daily. After this treatment, she was disease free for one year without administration of steroids (Figure 2, Panel D).

## Discussion

Subglottitis is an inflammation occurring in the subglottic area, which results in airway stenosis. Although mild cases only require watchful observation, in some cases, adrenaline and/or steroid inhalation may be needed. Advanced cases require oral or intravenous administration of antibiotics and steroids. Severe cases require urgent airway management, including intubation and tracheotomy. As subglottic stenosis shows rapid progression, an investigation of accurate etiology is important.

BD is a systemic inflammatory disease characterized by widespread inflammation of blood vessels throughout the body [4-7]. Four main symptoms, namely, oral ulcers, skin lesions, ocular involvement, and genital ulcers, are well known. BD can affect various systems such as the nervous, cardiovascular, and respiratory organs [4,7-13]. Although the cause is unknown, genetic predisposition and some external factors are thought to play a role in the pathogenesis of the disease [4,6]. Association between BD and human leukocyte antigen B51 (HLA-B51) has been reported; however, HLA-B51 was negative in this case [14].

Oral ulcers are the most common symptom. As they are often painful, they are a common initial symptom of BD. Pharyngeal and laryngeal lesions associated with BD have been reported, and some cases have shown pharyngolaryngeal stenosis [11-13]. As a recurrent and inflammatory disease, surgical treatment may be required in cases of pharyngeal stenosis caused by scarring or adhesions [13,15]. Voice disorders without obvious fiberscopic findings in the pharynx have also been reported, and vasculitis appears to be one of the underlying causes [16]. However, to our knowledge, no reports of concomitant subglottic stenosis caused by BD have been reported.

In this case, the diagnosis of BD was difficult at first presentation because typical manifestations of BD, such as ocular lesions or oral sores, were not observed [8,9]. Subglottitis is usually caused by viral and/or bacterial infection. In this case, the presence of cutaneous manifestations suggested a specific systemic disease, leading to the diagnosis of BD. In addition, subglottic laryngitis was accompanied by mucosal ulceration. Although there are no reports of BD occurring in subglottitis, mucosal ulcers are characteristic of BD [17]. Therefore, it is important to suspect BD when mucosal ulcers are observed. Apremilast, a selective inhibitor of the enzyme phosphodiesterase 4, is used in psoriasis and psoriatic arthritis, but is also effective in BD [18]. The long-term use of corticosteroids may result in an immunocompromised state [19]. In this case, apremilast was also effective in BD, and the recurrence of dyspnea was prevented. As a result, she was spared from continuing corticosteroids.

## Conclusions

In our experience, BD may cause subglottitis and subsequent respiratory distress at initial presentation. Subglottitis itself is a relatively common condition caused by infection, but it may develop as part of a systemic disease. Laryngeal fiberscopy is important for the diagnosis of acute dyspnea, but assessment of latent etiology is essential.
